# Socio-culturally mediated factors and lower level of education are the main influencers of functional cervical cancer literacy among women in Mayuge, Eastern Uganda

**DOI:** 10.3332/ecancer.2020.1004

**Published:** 2020-01-21

**Authors:** Alfred Jatho, Maniple Everd Bikaitwoha, Noleb Mugume Mugisha

**Affiliations:** 1 Uganda Cancer Institute, PO Box 3935, Kampala, Uganda; 2 Uganda Martyrs University, PO Box 5498, Kampala, Uganda; 3 Department of Cancer Control and Population Health, National Cancer Centre Graduate School of Cancer Science and Policy, Goyang, South Korea

**Keywords:** cervical cancer, functional health literacy, print literacy, oral literacy, numeral literacy, e-health literacy, perceptions, enablers, nurturers, existential beliefs

## Abstract

**Background:**

Health literacy (HL) is the degree of an individual’s knowledge and capacity to seek, understand and use health information to make decisions on one’s health, yet information on the functional level of cervical cancer literacy in Mayuge and Uganda as a whole is lacking. We, therefore, assessed the level of functional cervical cancer literacy among women aged 18–65 years in Mayuge district in five functional HL domains; prior knowledge, oral, print, numeracy and e-health. Understanding the factors associated with cervical cancer literacy is also pertinent to cervical health communication programming, however, no study has documented this in Uganda and particularly in Mayuge. Mayuge is a rural population based cancer registry and one of the sites for piloting cancer control interventions in Uganda. We also assessed the factors associated with cervical cancer literacy and awareness about currently available cervical cancer preventive services.

**Methods:**

The study protocol was approved by the Uganda Cancer Institute research and ethic committee (UCI-REC). In August 2017, we assessed five HL domains; cervical cancer knowledge, print literacy, oral literacy using audio-clip, numeral literacy and perceived e-HL among 400 women at household levels. Correct response was scored 1 and incorrect response was scored 0 to generate the mean percentage score for each domain. The mean scores were classified as limited, basic and proficient bands based on the McCormack HL cut-offs scale for knowledge, print, oral and e-health and Weiss cut-offs in the newest vital signs (NVS) for numeracy. We used the cervical cancer literacy scores to explore the effect of selected study variables on cervical cancer literacy. We also conducted five focus group discussions (FGDs) based on the theoretical constructs of the PEN-3 model.

**Results:**

The majority (96.8%) of the participants demonstrated a limited level of cervical cancer literacy with a mean score of 42%. Women who had completed a primary level of education or lower (OR = 3.91; p = 0.044) were more likely to have limited cervical cancer literacy. The qualitative data indicated that the women had limited cervical cancer literacy coupled with limited decisional, social and financial support from their male partners with overall low locus of control. Most (92.3%) of the women were not aware of the available cervical cancer services and had no intention to screen (52.5%).

**Conclusions:**

The women in Mayuge in general have limited cervical cancer literacy except oral HL domain. Limited cervical cancer literacy was highest among women with lower level of education and overall literacy seemed to be influenced on the higher side by socio-cultural constructs characterised by limited decisional, social and personal resources among the women with overall low locus of control. The Mayuge women further demonstrated scant knowledge about the available health services in their district and low intention to screen. Multi-strategy cervical health empowerment programme is needed to improve cervical HL using orally disseminated messages.

## Introduction

Cancer is the leading cause of death worldwide with 8.2 million deaths [1] and this is expected to increase [2], with up to three quarters of all cancer deaths by the year 2050 [1] occurring in developing countries. Cancer of the cervix is the commonest type of cancer in Uganda with 80% of cases at late stage of the disease [3].

Kampala Cancer Registry shows an increase in the incidence of cervical cancer by 1.8% [4]. Health literacy (HL) means the individual’s capacity to seek, understand and use health information to make decisions on one’s health [5, 6, 7]. Viewed as cognitive and social skills, HL can influence the motivation and ability of individuals to gain access to health information, and to understand, promote and maintain health [8]. A higher level of cancer literacy is known to improve cancer preventive health [7, 9], consequently, the critical factors associated with cervical cancer literacy level need to be identified and prioritised.

Diviani *et al* [7] and Nutbeam [6] defined three levels of HL: functional HL (FHL), which denotes basic cognitive ability (reading and writing) that helps individuals to understand health information, interactive HL, which denotes advanced cognitive and social skills that enable individuals to take an active role in their health-related decisions and lastly critical HL which refers to more advanced cognitive abilities that enable individuals to appraise health information and advice critically and take the appropriate health decision. This implies that a good cancer literacy evaluation should consider all three levels of HL. Most studies on HL concentrate on print literacy, that is reading health messages with comprehension [10, 5].

HL in terms of functional, interactive and critical HL is known to help individuals to understand health information, take an active role in their health-related decisions and enable them to appraise health information and advice critically and take the appropriate health decision, respectively [6]. People’s level of cancer literacy is evidenced to influence their respective cancer preventive health behaviours [7, 11].

Han *et al* [12] in a cluster-randomised trial of cervical and breast cancer screening literacy intervention in Korean women led by community health workers, demonstrated that cancer screening and other cancer preventive behaviours were successfully promoted when they were HL focused, therefore, consideration for the individual and population HL level is coupled with evidence-based cervical health communication.

A qualitative study [13] on beliefs and perceptions among women about cervical cancer using focus group discussions (FGDs) found that cervical cancer and other cancers are not well understood, especially in rural Ugandan communities. For instance, there are various beliefs and misperceptions among Ugandan women about cervical cancer. Some people believe it is an incurable disease, a disease from men, a disease whose cause is unknown and human papilloma virus (HPV) vaccination as a form of family planning.

According to Gaglio *et al* [14] and Giuse *et al* [15] most people of any literacy level prefer and are able to receive cancer information during face-to-face interactions with health workers. However, the health messages need to be culturally appropriate [16] to benefit the targeted population. This means cervical health information should preferably be orally disseminated by health workers in a culturally sensitive way. However, there is a growing tendency for production and demand of health messages in print formats like flyers and brochures among others as well as electronic messages. What is not known is whether a sizeable proportion of our Ugandan population is capable of reading this information with comprehension.

Since the level of cancer literacy is known to improve cancer preventive health behaviours, it should not be compromised in any way [17, 5]. Cancer literacy constitutes general knowledge about cancer risk factors, diagnosis and treatment, numeracy, print, oral and e-HL. These are important cancer health message areas that an individual needs to know [18].

## Existing HL assessment tools and their gaps

Several HL assessment instruments have been developed to evaluate the HL skills of individuals; however, these instruments differ in designs, approaches and modes of administration to respondents and the population setting differentials. The five commonest groups of tools applied in HL metrics are: 1) the rapid estimate of adults’ literacy in medicines (REALM) and the test of FHL of adults (TOFHLA); 2) the e-HL scale (eHEALS); 3) the newest vital signs (NVS) tool; 4) the cancer message literacy test – listening (CMLT – listening) and 5) HL skills instrument (HLSI) and European HL survey questionnaires (HLS-EU-Q).

### 1) The REALM and the TOFHLA

The REALM was developed to measure reading ability and pronunciation of medical information [19]. The TOFHLA was developed to measure an individual’s ability to read and understand health information [19]. Both REALM and TOFHLA are limited to reading ability, yet awareness (prior knowledge), numerical and listening ability are also key elements of HL. However, both are easy to implement among individuals with reading ability. Individuals with lower print cancer literacy benefit less from printed health information such as flyers, magazines and pamphlets that are commonly provided in healthcare facilities [20].

### 2) The eHEALS: assessing interest in electronic health resources

The eHEALS [21] which is a five-point Likert scale was developed for self – evaluation of an individuals’ ability to obtain, evaluate and use electronic health information to decide and address their health needs. The original e-health tool consisted of eight items; however, Norman and Skinner added two additional items for assessing interest in electronic health resources. This e-health tool comprised of ten items rated on a five-point Likert scale and was adapted for the e-HL domain of this study.

The United States’ national cancer institute (NCI) ‘Health Information Trends Survey’ of 2010 showed that younger people used the internet to seek health information more than adults and consequently had a higher level of HL [22]. Therefore, e-HL is a crucial aspect of cancer literacy [23]. The use of phones, including internet enabled phones, especially smart phones, has rapidly increased in Uganda. Phones and other internet enabled devices can enable provision of information on health behaviour, cancer prevention, diagnosis and treatment. In line with phone usage, the Uganda communication commission of 2014 in its country-wide study on mobile phone access and usage in Uganda indicated that at least 52.3 % of households in Uganda had access and were using mobile phones.

Since Telephone subscribers in Uganda rose from 16.7 million in 2012 to 18.3 million in 2013 and internet rose by 33.6% from 2,692,705 in 2012 to 3,625,559 in 2013 as reported by Uganda communication commission in 2015, use of mobile phone message platforms for cervical cancer health information dissemination to the public can leverage this opportunity. In addition to the 73% and 69% of self-reported household mobile phone use and mobile phone ownership, respectively; 31% of this being feature and smartphone [24], the ability to benefit from phones to receive and search for cancer information needed to be assessed.

### 3) The NVS tool: numeracy HL skills

The NVS tool was developed to measure health numeracy skills and ability to infer on health information in health facility settings [25]. This lacks the print (reading), oral (listening) and the e-health domains of HL. It is vital for people to use their cancer numeracy skills when deciding on benefits and risk of certain health behaviours such as tobacco use, excess alcohol intake, cancer screening and compliance with treatment recommendations [26]. People with lower cancer-related health numeracy are more likely to experience a lower health status [27].

Cancer-related health numeracy skill is more critical for the prevention and management of chronic diseases such as cancer [14]. For instance, Kaplan *et al* [28] found that women who had higher health numeracy scores were more willing to uptake breast cancer chemoprevention drugs compared with women with lower health numeracy scores.

Since this study included only those who could read and write for the quantitative phase, it was possible to assess cancer-related health numeracy skills among the study participants.

### 4) The CMLT – listening

CMLT was developed to evaluate oral cancer literacy, which is listening with comprehension of spoken cancer messages [11, 29]. This lacks the numeracy, print and the e-health domains of cancer literacy.

In practice, most people of any literacy level prefer and are able to receive cancer information during face-to-face interactions with health workers [14, 15]. There are few studies about the impact of oral HL on health behaviours. Previous studies indicated that individuals with low oral HL skills have an increased risk of not understanding spoken health messages and hence mismanage their health concern. For instance, Lyratzopoulos *et al* [30] found that the extent to which individuals extract meaning from spoken health information can affect their ability to act on healthcare facility information such as hospital discharge instructions.

### 5) HLSI and HLS-EU-Q

In an attempt to bridge the gaps between the different HL instruments, especially limited dimensionality of assessing HL comprehensively, the institute of medicine (IOM) made a call to action in the HL report titled ‘a prescription to end confusion’ [31].

In response, McCormack [32] developed a 25-item HLSI and a short version 10-item instrument (HLSF-SF) that attempts to address the gaps left by other HL tools. HLSI assesses the four domains; print, oral, numeracy and e-HL. However, this tool has some limitations such as computer based and email modes of administration that are difficult to apply at the moment in a rural Ugandan setting like Mayuge district. However, it is easy to analyse and interpret the findings as the scores are reported in percent of correct responses.

Sorensen *et al* [33] developed a tool for measuring HL in a population entitled HLS-EU-Q. This tool was reported by many researchers as being a comprehensive and innovative tool for assessing perceived HL in populations, other than other tools that were oriented more towards clinical settings [34]. To stimulate wide application of HLS-EU-Q, it needs to be validated in many countries to improve the understanding of HL in varying populations [33]. However, this tool measures the perceived HL, without psychometric evaluation of the print, oral, numeracy and prior health knowledge of the individuals.

According to Diviani and Schulz [18] cancer literacy includes general knowledge on cancer risk factors, diagnosis and treatment in addition to numeracy, print, oral and e-HL. The general knowledge can therefore be assessed based on the constructs and subconstructs in the Diviani and Schulz conceptual definition of cancer literacy.

As observed above, most of these HL tools did not cover the full breadth of notions embodied in the conceptual definition of HL [35]. In addition, the tools had substantive psychometric deficiencies [35]. Psychometric measurement is paramount in HL evaluation to assess knowledge, skills and attitudes and perceptions wherever applicable.

One of the weaknesses of existing HL studies is the reliance on measurement of FHL, that is, an individual’s ability to read and understand medical information, these do not assess the complexity of the HL concept, and therefore, little or nothing can be deduced on the effect of other dimensions of HL in understanding human health behaviour [5].

According to McCormack [32], a more holistic approach to understand HL requires assessment of four separate measurable domains of HL: effective listening and speaking (oral literacy), ability to read and understand health information (print literacy), use and understanding of quantitative health information (numeracy literacy) ability to search and navigate health information through the internet (e-HL).

The functional cancer literacy assessment was, therefore, based on the constructs and subconstructs in the Diviani and Schulz conceptual definition of cancer literacy and the constructs of McCormack [36] pertaining message dimensions and what should a lay person know in the context of cervical cancer information. It was against this background that this study investigated the level of functional cervical cancer literacy among women aged 18–65 years in the Mayuge district by exploring various FHL domains.

It is crucial to note that the current cervical cancer control interventions in Uganda include public education during screening clinics, outreaches, workplaces, schools, places of worships like churches and mosques and mass media like radio-talk shows, screening in facilities and in community outreaches and treatment of precancer and cervical cancer disease at the Uganda Cancer Institute. The HPV vaccine targeting girls aged 10 years old was introduced in 2014 and rolled out nationally by 2015 as part of the routine immunisation programme.

Similarly, for clarity, Mayuge is a newly established rural population cancer registry in Eastern Uganda. Mayuge district is the first ever rural population cancer registry in Uganda at Kigandalo Health Centre IV in Mayuge district for cancer registration and modelling interventions, thus the need for baseline cancer literacy information. Cervical cancer being the commonest cancer in Uganda and the top cause of cancer death in Ugandan women, it is, therefore, taken as a flagship for HL assessment.

The current gap in cancer HL metrics, especially the lack of data on cervical cancer literacy level and the need of understanding the population performance in the various FHL domains warranted a multi-dimensional HL evaluation. We assessed five FHL domains: 1) cervical cancer prior knowledge to assess cervical cancer awareness level; 2) print literacy to assess capacity to read cervical health key messages with comprehension; 3) oral literacy using pre-recorded audio-clip to assess capacity to listen to orally disseminated key cervical health messages with comprehension; 4) numeral literacy to assess capacity to understand numerical data in cervical health messages and 5) perceived e-HL to assess interest and ability to use electronic health information on mobile phone platform.

## Factors that are known to influence HL level

Diviani and Schulz [7] revealed that level of education was linked to cancer literacy. In subjective contexts, the perceived prevalence of a disease risk is a potential motivator of health promoting behaviour that can facilitate better utilisation of cancer prevention, detection and control services [37, 38]. Hasahya *et al* [13] found that cervical cancer and other cancers are not well understood, especially in rural Ugandan communities. Some believe it is an incurable disease, a disease from men, a disease caused by family planning, or that the cause is unknown. Similarly, a study by Mwaka *et al* [39] found that the understanding of cervical cancer in Northern Uganda was driven by lay person perceptions. Therefore, pragmatic and in-depth exploration of the lead factors influencing cervical cancer literacy is important in cervical health promotion as a flagship cancer in Uganda.

What is well known from the evidence is that cancer of the cervix and its associated death can be prevented through effective screening and lifestyle modification. This is because precancerous lesions can be detected during screening 10 or more years before the cancer develops [40]. Late presentation probably occurs due to limited information about the risk behaviours and not being aware of the available cervical health services that can be sought for prevention and early detection purposes. In 2010, the Uganda Ministry of Health (MoH) and development partners in the health sector recommended and planned to roll out national cervical cancer preventive services including screening in both regional and district health facilities as contained in the MoH cervical cancer strategic plan of 2010/11–2015/16. However, there is general undocumented concern of limited awareness and access to cervical cancer services as a flagship including screening.

This study primarily assessed the functional level of cervical cancer literacy and the factors influencing cervical cancer literacy as flagship cancer in Uganda. We also assessed whether rural Ugandan women in Mayuge are aware of the currently available cervical cancer preventive services in their district health facilities and regional hospitals and to describe the cervical cancer-related avoidance practices (or risk behaviours) among women in the Mayuge rural cancer registry population from the study participants’ perspectives to inform future programme planning in the Mayuge rural population.

## Methods

### Research design

This was a population-based cross-sectional concurrent mixed methods design [41]. A structured questionnaire was used to collect quantitative data from 400 women at household levels and five FGDs were used to collect qualitative data based on the theoretical constructs of PEN-3 model. The qualitative strand using the FGD refined, explained and explored in-depth to give the essence [41]. The umbrella paradigm of this design was pragmatism [42]. We used the cervical cancer literacy score that was assessed on the same participants to explore the effect of the selected study variables.

## Inclusion and exclusion criteria

*Inclusion criteria*: Women aged 18–65 years who could read and write in either English or Lusoga (the main local language) or both with no hearing and vision impairment because this study included assessment of reading and listening with comprehension. The participant must have been a resident of Mayuge for at least 2 years to provide fairly correct information about the population.

*Exclusion criteria*: Individuals with documented or reported hospital diagnosis for cervical cancer and those whose spouses or first-degree relatives had documented or reported diagnosis of cervical cancer were excluded because they are more likely to have better prior knowledge about cervical cancer than others.

*Sample size*: Sample size for the quantitative strand was computed using Cochran formula (1963:75) since the population size was large, using *n* = *Z*^2^*pq*/*e*^2^ where; *n* = desired sample size, *Z* = the abscissa of the normal curve that cuts off an area α at the tails (standard normal deviate) corresponding to 95% confidence interval (CI) = 1.96, *p* = the proportion of 50% = 0.5 (maximum variability) was used because no proportion was found in literature for similar population, *q* = 1 −* p*, *e* = precision of 5% to allow a 95% interval around the estimates was used. Substituting in the above formula gives a sample size of 385 = 400 study participants (increased for contingency). Sample size for FGD was based on saturation as advised in the 2014 research designs by Creswell.

## Study setting

This study was conducted in Mayuge rural cancer registry population located in the Eastern region of Uganda. We enrolled 400 adult women aged 18–65 years in Mayuge.

## Sampling technique

A multistage cluster sampling was used where the two counties of Bunya South and Bunya East were selected using a simple random sampling by a raffle from the three counties of Mayuge district–Bunya West, Bunya South and Bunya East. Thereafter, a systematic random sampling approach was used to select 40 villages from the two counties, that is, 16 out of 142 villages in Bunya East and 24 out of 225 villages in Bunya West.

Village selection was based on allocation by proportion to size and every ninth village in Bunya East and every ninth village in Bunya West from the respective county list of villages listed by sub counties was selected. It should thus be understood that each selected village from the village list was a cluster and from each cluster, ten households from the village household list were selected by systematic random sampling from the village household list. In each selected household, one eligible participant was selected using the Kish Grid table of random numbers for participant’s selection to remove selection bias [43, 44].

## Data collection instruments and procedures

Quantitative data were collected using a structured pre-tested questionnaire designed in English and translated to Lusoga (the indigenous local language spoken in Mayuge) following rigorous refinement by the Uganda Cancer Institute Research in progress panel. Participants read and answered questions on prior knowledge, print messages, messages with numeral information and e-health perception. On oral HL domain, a pre-recorded cervical cancer audio message was played once and participants were asked (using predetermined questions in the questionnaire) to answer questions relating to what was communicated in the audio messages.

Qualitative data from five FGDs of eight adult female participants each (totalling to 40 participants) based on PEN-3 Model was generated. PEN-3 Model stands for Perceptions–Enablers–Nurturers, Positive–Existential–Negative and Person–Extended Family–Neighborhood [45, 46]. The PEN-3 model comprises of three domains; relationship and expectations, cultural empowerment and cultural identity. Each domain is divided into the PEN acronym: Perceptions–Enablers–Nurturers, Positive–Existential–Negative and Person–Extended Family–Neighbourhood [47].

The relationships and expectations domain were used to identify perceptions, enablers and nurturers that improve or act as a barrier to cancer literacy. The cultural empowerment domain was used to identify the positive, existential and negative beliefs, values and relationships that relate to cancer literacy. The cultural identity domain was not used because it is used to establish whether the intervention target entry point should be the person, the extended family or neighbourhood [47].

## Quality control

*Ethical approval*: This study was first approved by Uganda Martyrs University and thereafter approved by the Uganda Cancer Institute Research and Ethic Committee (UCI-REC).

*Training Research Assistants*: Research assistants underwent two days of orientation training on the purpose and procedures of the study, including the sampling procedure. The research assistants were closely supervised by the principal researcher during data collection.

*Pre-testing the cancer literacy tools*: The questionnaire was pretested on 20 participants in the non-sampled county of Bunya west in Mayuge and refined before the main study was undertaken.

## Data analysis

Data entry was done in Excel and later exported and analysed using IBM SPSS version 20 statistical application. The cut-off levels indicating adequate (proficient), basic (average) and limited (poor) were considered as the mean scores in relation to cervical cancer literacy levels of the participants. Every correct response was scored 1 point and every incorrect response was scored 0 points. The mean scores were classified as limited, basic and proficient bands based on the McCormack HL cut-offs scale for knowledge, print, oral and e-health and Weiss cut-offs in the NVS for numeracy.

The HL level cut-offs (classification) based on McCormack *et al* [9] cut-offs is classified as: Adequate is ≥ 82, Basic is 70–81 and limited is <70 for prior knowledge (awareness), oral, print, perceived e-health. Weiss *et al’*s [25] cut-offs in the NVS for numeracy are classified as: Adequate = 4–6 correct responses, Basic/low = 2–3 correct responses and Limited = 0–1 correct response. In the perceived e-health domain, the statement agrees, strongly or agrees and useful or important and very useful were scored as 1 and the rest as 0 to generate the score for perceived e-health.

The relationship between the literacy band score and independent variables was determined using Pearson’s chi-squared test or Fisher’s exact test (for cell counts less than five counts) to report the relationships between the independent variables and the literacy score. The qualitative data from five FGDs was analysed using qualitative content manifest analysis (surface structure) to report themes and draw interpretation of the findings. The findings were categorised into positive, existential and negative cancer beliefs and values under the cultural empowerment domain while the relationships and expectations domain were categorised into perceptions, enablers and nurturers.

## Results

### Demographic characteristics

Out of the 400 women who participated in this study, most (33.8%) were aged 18–25 years with mean age of 32.9 years, most (76.3%) were either married or cohabiting, the majority (53.0%) had completed primary level of education (Table 1).

## Level of cervical cancer literacy among women aged 18–65 years in Mayuge district

The average scores of cervical cancer literacy categorising the participants into two literacy bands of limited and unlimited (basic and adequate levels) obtained on the same participants at the same time is presented in Table 2a and 2b. Out of the 400 participants, most (90.5%) of the women had limited knowledge about cervical cancer in terms of risk factors and prevention, early detection and treatment with an average score of 36.3% (SD = 21.2). The study further shows that most (77.5%) of the women were characterised by limited cervical cancer print literacy in terms of reading cervical cancer information with comprehension, with an average score of 51.8% (SD = 24.1). The study also reveals that most of the women (98.5%) in Mayuge district were characterised by limited cervical cancer-related numeral literacy (understanding numerical data in cervical health messages) with an average score of 27.1% (SD = 22.3). The majority (90.3%) had limited cervical cancer e-HL in terms of interest and capacity to use electronic health information, especially mobile phone messaging platform with average score of 23.7% (SD = 26.9). However, most (63.1% in basic and average bands) of the participants demonstrated basic (average) level of cervical cancer oral HL, that is, listening with comprehension of orally disseminated health messages with an average score of 74.4% (SD = 23.6)**.** The composite results showed that most (96.8%) of the women had a limited level of cervical cancer literacy with an overall mean score of 42.7% (SD = 12).

## Factors associated with cervical cancer literacy among women aged 18–65 years

Table 3 shows that the proportion of women with limited cervical cancer literacy was highest (98.6%) amongst those who had stopped at primary level of education compared to women who had studied beyond the primary education level. This difference in proportions of mothers with limited cervical cancer literacy was found to be statistically significant (*p* = 0.044 < 0.05). This meant that cervical cancer literacy is significantly influenced by the education level of the women. There was no marked difference on the proportion of women with limited cervical cancer literacy with respect to radio ownership; those who owned a radio (95.5%) and those who did not own a radio (100.0%) were both in the limited literacy band, yet the variation in proportions of women with limited cervical cancer literacy was statistically significant (*p* = 0.024 < 0.05). This was also observed among women who had visited health facilities during the last 12 months versus the women who did not visit health facilities during the last 12 months, yet the variation in proportions of those with limited cervical cancer literacy was statistically significant (*p* = 0.025 < 0.05).

The study, however, varied in the sense that being screened for cervical cancer was not significantly associated with cervical cancer literacy in addition to other factors such as intention to screen, HPV vaccination availability in the nearby health facility, age, income, occupation and language. This implied that age, income, occupation and language alongside being screened for cervical cancer are not significant predictors of cervical cancer literacy among the women in Mayuge district, except education level, health facility visits and radio ownership.

Findings relating to the personal factors which were thought to be insignificant showed that limited cervical cancer literacy was highest amongst women who perceived their risk of developing cervical cancer as low and lowest among women who perceived their risk of developing cervical cancer as high (*p* = 0.617 > 0.05). However, the perceived risk of cervical cancer development is not a significant predictor of cervical cancer literacy among the women in Mayuge district.

The foregoing results were, however, collaborated with qualitative results from the FGDs as shown in Table 4 below.

## Qualitative results: the socio-economic and cultural factors related to cervical cancer literacy

The ability to obtain basic cervical cancer information and services required to make appropriate health decisions was assessed qualitatively using FGD and the results were analysed using manifest content analysis; the emerging themes were classified based on the PEN-3 model (Table 3). This was aimed at providing either contradictory or corroborative evidence relative to the quantitative results of cervical cancer literacy.

The FGD participants were asked ‘what comes to your mind when you hear the word cervical cancer’. The discussion yielded the following responses:

*You cannot survive from it because it has no cure yet* (FGD1, participant 5). This indicates a limited level of cervical cancer literacy

When asked what you think causes cervical cancer, the responses were as follows:

*The main cause of cancer in woman is use of family planning, the health workers should first test these family planning drugs before they give us- they should be having cancer in them. We used not to hear of cancer like these days* (FGD2, participant 7). This was said by eight women out of the thirty women who participated in the three FGDs (ten women per FGD). Therefore, the participants perceived use of modern family planning, miscarriage and poor personal hygiene as being the risk factors of cervical cancer.

When asked what you think are some of the things that can stop you from getting any of the cervical cancer screening tests that you may be eligible for, the participants mentioned that:

*No place for us to test and treat cancer near Mayuge here, so what can we do? - just to wait for death when it comes to cancer.* (FGD2, participant 6)

*My sister went to Mulago when she was referred form Iganga district hospital, she found many people in line waiting to be checked and failed to be tested on that day- she slept there....... and was tested on next day and told to pick the result of the sample removed after one week! You see the problem; how do you think everybody especially for us in the rural villages here to afford that. For my sister she managed to go there because her husband is a secondary school teacher who gave her the money.* (FGD2, participant 4)

This demonstrated that a lack of cervical cancer screening services in Mayuge district, lack of money to access services, long distance to Kampala, long waiting time and delay of test results at Mulago-Uganda cancer institute were the main things that hinder them from accessing cervical cancer preventive and early detection services.

When asked how did you learn about cervical cancer, the participants narrated the following: *Health workers mainly talk about malaria, HIV/AIDS, antenatal care and family planning- they don’t educate us on cancer- if the disease is cancer they tell people to go and check from Mulago* (FGD2, participant 1).* We normally once in a while hear about cancer over the radios from Jinja* (FGD2, participant 2). This shows that their health facilities mainly conduct health talk on other diseases like HIV/AIDS, not cancer, meaning they lack opportunities for cancer awareness, however, some noted that once in a while they get some cancer information from their local radio stations.

When asked what factors are likely to influence their ability to obtain and understand cervical cancer information and services, most of the participants responded as quoted:

*Of course not only the lack of money, like for me with six young children how do you think your husband will allow you to go for cancer checkup and you leave him with the children at home- it is impossible!- at least if it was in our nearby hospital you can hurry and come back home in time to cook.* (FGD1, participant 5)

*The radio announcements and sensitization about health are usually aired faster at times when we are cooking or in garden so you miss many things.* (FGD1, participant 8)

*We cannot get the cancer information and services because the health workers do not educate us about cancer* (FGD1, participant 3). These point to low level of income, lack of cancer information at health facilities, poor timing and rapid speech in airing of health talks from radios as the main concerns that influence the ability of the women to obtain and understand cervical cancer information and services.

When asked what factors are likely to influence your ability to decide to lead a healthy lifestyle that can reduce the risk of developing cervical cancer, the participants narrated their concerns as below:

*Poverty is big problem here …. You can’t manage to be healthy when you don’t have money to eat good food.* (FGD3, participant 7)

*You cannot decide alone, you must agree with your husband first, because he is the leader of the household.* (FGD3, participant 3)

*People say that the test for cancer of the uterus can make a woman infertile, so some of us people fear it – mainly those who have many children and those who are strong hearted who can dare for it* (FGD1, participant 6). Therefore, poverty was their biggest challenge towards maintaining a healthy lifestyle.

## Knowledge of cervical cancer preventive services available in Mayuge district

Most (92.3%) of the women were found to be unaware of any routine cervical cancer screening in Mayuge district. The majority (95.8%) of the women did not know that there are cervical and other cancer-related health education services and 70.0% were not aware of sexual and reproductive health services in the nearest health facilities in Mayuge district.

The results show that most (64.0%) women were unaware of either the provision of free condoms through village health teams (VHTs) or HPV Vaccination (67.5%) for 10 year old girls in Mayuge district. The results however, show that most women know that there was HIV Counselling and testing (87.3%), provision of free condoms at the health facility (88.5%) and antiretroviral treatment (ART) services in the nearest health facilities in Mayuge district (79.3%) (Table 5).

## Cervical cancer-related risk behaviours

Most of the women had never been screened for cervical cancer (76.0%), or had intention to screen (55.8%) yet were characterised by a history of sexually transmitted infections (STIs) other than HIV (50.5%). Most of the women who participated in the study had had multiple lifetime sexual partners (96.3%) and started having their first sexual intercourse at less than 18 years of age (85.8%).

The results showed that most of the women had their first pregnancy at an age less than 20 years (82.3%), never using condoms (60.8%) moreover with parity of three children and above (77.0%). In addition, none of the women that participated in the study had been vaccinated against HPV (0.0%). Fortunately, most participants had no history of tobacco use (93.0%), no history of alcohol consumption (65.5%), and no history of sexual intercourse for financial gain (91.8%) (Table 6).

## Discussion

### The level of functional cervical cancer literacy

The findings indicate that women in Mayuge lack the knowledge and capacity needed to seek, understand and use cervical cancer-related health information to make decisions on one’s health. However, the women in Mayuge district demonstrated a basic level of oral cervical cancer HL, implying that they have the ability to listen to orally disseminated (spoken) health messages with comprehension unlike other health communication domains.

The prior knowledge literacy level was low among women in Mayuge. This result equally compares well with those by Hasahya *et al* [13], which found that cervical cancer and other cancers are not well understood, especially in rural Ugandan communities. For instance, there are various beliefs and misperceptions among Ugandan women on cervical cancer: some believe it is an incurable disease, a disease from men, a disease whose cause is unknown and in HPV vaccination as a form of family planning.

There was also concurrence with a study by Mwaka *et al* [48] who found that, despite cervical cancer being the most common cancer affecting women in the Acholi subregion of Northern Uganda, the community understanding of the cervical cancer disease was limited. Therefore, there is a need for cervical health information dissemination in Mayuge to improve the awareness component of FHL.

In this study, oral HL scores were quite high, thus indicating that the Mayuge women are able to listen with comprehension to spoken or audio cervical health messages, be it from health workers or radios. This agrees with the findings of Gaglio *et al* [14] and Giuse *et al* [15] that most people of any literacy level prefer and are able to receive cancer information during face-to-face interactions with health workers and are likely to understand it better compared to other means of communication. This means that health communication programming should take note of the limited literacy level in Mayuge district, and prioritise health messages that are orally encoded to the community since there was a fair score in the oral literacy component.

Furthermore, concerted cervical cancer awareness campaigns to improve cervical HL, and promote prevention and early detection need to be culturally sensitive and context-specific, and include messages on risk factors, symptoms and treatment in a way that corroborates with what Sanders *et al* [16] reiterated – that culturally appropriate health communication provides an important opportunity to tackle health disparities.

In this study, the authors argue that health practitioners and policy makers can use these current findings in adopting strategies towards improving cervical cancer literacy in the domains of prior knowledge, print, numeral and e-health where the participants demonstrated difficulty in comprehending cervical cancer-related information, and therefore, apply the knowledge to improve cancer prevention and control interventions to promote healthier lives while leveraging on orally disseminated cervical cancer information where the participants demonstrated a fairly average level of cervical cancer literacy.

Despite the increased ownership and usage of mobile phones in Ugandan households and the expected benefit in health information dissemination [23, 24, 49], the perceived interest and ability of using phones to access health messages was low in terms of e-HL score. This implies that women in Mayuge district are less likely to benefit from the mobile phone health messaging applications. Therefore, when planning to use such applications, improving the individual capacity to navigate the phone messaging platform and motivation for its benefits should be part of the products when launching such application.

Uganda Cancer Institute (UCI) can apply this result as a baseline to examine the influence of cancer-related health services within the region on rural health service delivery and specific improvements that could be made to the health system to meet the needs of the rural community and healthcare providers. It is also argued that since the lack of knowledge on HL levels denies health promoters a chance to effectively educate the public to promote cervical health, cervical cancer communication can, therefore be tailored to effective health education and health information materials to the current cervical cancer literacy level.

Additionally, cancer education can be integrated into the school curriculum and in school health clubs like the children caring about cancer (3Cs) club in Uganda – an initiative by the Uganda Cancer Institute to increase cancer awareness in schools and the surrounding communities, alongside training health workers on HL improvement.

## Factors influencing cervical cancer literacy

Cervical cancer literacy was found to be significantly influenced by the education level of the women. Such findings are similar to those found by Diviani and Schulz [7] which revealed that level of education was linked to cancer literacy. The results equally found personal and family resources such as radio ownership to be statistically significant, despite non-remarkable effect size. The study, however, found that limited cervical cancer literacy was not statistically significant though highest amongst women who perceived their risk of cervical cancer development as low and lowest among women who perceived their risk of cervical cancer development as high. Such results were not comparable to earlier studies that showed that perceived prevalence of a disease risk is a potential motivator of health-promoting behaviour that can facilitate better utilisation of cancer prevention, detection and control services [37, 38].

The qualitative data derived from application of the PEN-3 theoretical model indicated that the women had limited cervical cancer literacy coupled with limited decisional, social and financial support from their male partners with overall low locus of control. These results were comparable to earlier results by Airhihenbuwa and Okoro [47] and Iwelunmora Newsomeb and Airhihenbuwa [46] constructs of the PEN-3 theoretical model that cultural empowerment, relationships and expectations influence ability of individuals to seek, understand, appraise and use health information to decide and practice healthy behaviour and that it is recommended to use theory-based health messages [50]. These results equally compare well with those by Hasahya *et al* [13] that found that cervical cancer and other cancers are not well understood, especially in rural Ugandan communities. Some believe it is an incurable disease, a disease from men, a disease caused by family planning, cause is unknown, and HPV vaccination as a form of family planning. There was also concurrence with a study by Mwaka *et al* [39] who found that the understanding of cervical cancer in Northern Uganda was driven by lay person perceptions.

The results, in addition, fail to compare well with earlier studies that people’s beliefs about the likelihood of developing cancer (perceived cancer risk) influence their decision on actions that reduce cancer risk. Many studies showed that individual’s actual cancer risk (risk score based on health workers’ assessment may differ from that perceived by the individual [51–53]. Perceived cancer risk is, therefore, a subjective psychological event that relates to cancer threat, susceptibility and judgment as reported by Peipin *et al* [51].

It is beneficial to women when their cervical cancer risk is well estimated and informed of their risk when implementing cancer screening and risk reduction interventions, moreover some studies showed that most people do not perceive their cancer risk prevalence correctly [54]. Individual overestimation of cancer risk may lead to unnecessary anxiety, concern and over utilisation of cancer screening and other risk reduction options, while underestimation may lead to missed cancer screening and other risk reduction opportunities.

Similarly, Kartal *et al* [55] in a study to evaluate breast cancer risk perception of women attending health care services in Turkey found that most of the women (65.7%) in the high-risk group underestimated their risk of developing breast cancer. The researcher concluded that there was a need to understand this optimism. This means that enhancing our understanding of individuals’ perceived cancer risk prevalence and factors associated with cervical cancer literacy could guide the development of a culturally relevant health-promoting communication approach that contributes to the reduction of the cervical cancer burden.

## Awareness of available services and risk reduction behaviour

Regarding awareness about cervical health services, this study indicated unawareness about the availability of services like cervical cancer screening, cervical cancer and other cancer-related health education alongside sexual and reproductive health services in the nearest health facilities. These results call for improvement in accessibility to cancer-related preventive and care services countrywide through establishing mobile cancer and continuity clinics (MCCCs) as per the recommendations by the Uganda MoH report (2014), equipping and building the capacity of health workers at regional hospitals and district health facilities.

The results further show that most women neither know about the provision of free condoms through VHTs nor HPV vaccination for girls 10 years old or in P4 class in Mayuge district. Such results correlate with results based on rural community experiences that a gap exists in population-based health innovation and service delivery, especially in rural areas [56]. However, the study found that most women knew that there was HIV counselling and testing, provision of free condoms and ART services at health centres. Such results are different from those earlier found by Berkman *et al* [57] that indicated poorer knowledge of disease process and self-management skills, especially among individuals.

This study established that women in Mayuge district did not adhere to routine cervical cancer screening and cervical cancer and other cancer-related health education services in their health facilities. However, their cervical screening rate of 24% versus did not screen of 76% indicates improvement compared to the national cervical screening rate of 9.9% (6.9–12.8) that was reported by the Uganda MoH national non-communicable diseases (NCDs) survey of 2014. This increment in screening coverage could be attributed to cancer awareness and screening outreaches implemented nationally including in Mayuge by the Uganda Cancer Institute through its comprehensive community cancer programme (CCCP) unit. This is still below optimal level and compromises the prevention and early detection drives for cervical cancer in Mayuge district and other districts in Uganda with similar health system challenges.

These results relate well with earlier results in a study done in Vietnam that showed distance as a key determinant of patients’ delay to seek cancer services [58]. The demand barriers can be classified as ‘market failures’ and ‘pursuance of social equity’ [59]. Therefore, the MoH should ensure that district health facilities from at least health centre IVs and above provide cervical cancer screening services and all health facility levels from VHTs to national level to provide cervical cancer health education to their clients and catchment community- an entity of health promoting hospital/health promoting facility and community. The results correlate with what Bitarabeho [60] indicated that the decentralisation system of governance helps to effectively and efficiently deliver health services.

In this study it was also found that most women had intention to screen for cervical cancer. In regard to risky behaviours this study found that women in Mayuge district started having their first sexual intercourse at less than 18 years of age with most of those who participated having had their first pregnancy at an age less than 20 years, were characterised by a history of STIs other than HIV and had had multiple lifetime sexual partners. These results correlate with findings by Harding and Selman [61] which indicate inconsistency in health services provision and lifestyle risk exposures. Much results indicated neither instances of tobacco use, alcohol consumption nor sexual intercourse for financial gain, most women never used condoms, moreover with parity of three children and above and none of them had been vaccinated against HPV. This implies less use of what is equipped at the health centres within their district and low utilisation of cervical cancer screening services as put in the recommendations contained in the MoH 2010 cervical cancer strategic plan of 2010/11–2015/16.

## Conclusions

The women in Mayuge district have a limited level of cervical cancer literacy, meaning that they have limited knowledge of cervical cancer and low capacity to seek, understand and use cervical cancer-related health information to make decisions on their health. However, the women demonstrated a basic level of oral cervical cancer HL (ability to listen to spoken health messages with comprehension) unlike other domains. However, there is a need to assess cervical cancer literacy in all the major regions of Uganda to elicit the national outlook due to the socio-cultural and health services disparity. Health workers should prioritise use of orally disseminated cervical cancer information, use plain language with teach-back technique and conduct accelerated cervical cancer health education within health facilities, schools, places of worships and community settings to improve the prior knowledge component of HL.

The proportion of women with limited cervical cancer literacy was highest amongst those who had stopped at primary level of education (low level of education). Qualitatively, limited decisional, social and personal resources among women coupled with no or inadequate financial support from their male partners with overall low locus of control explain the low capacity of women in Mayuge to obtain and use cervical cancer preventive and early detection services.

The women in the Mayuge rural population cancer registry catchment currently have scant knowledge about the available cervical cancer preventive and control services in their district health facilities and regional hospital and are characterised by practices that are likely to increase the risk of developing cervical cancer. A multistrategy cervical health empowerment programme is, therefore, paramount. Health workers should put more effort into creating cervical cancer avoidance awareness in the community, schools and health facilities among other settings and promote the available cervical cancer-related preventive services in their district health facilities and regional hospital.

## Authors’ contributions

Jatho Alfred (Uganda Cancer Institute and Uganda Martyrs University) conceptualised and conducted the study. Prof. Dr Bikaitwoha Maniple Everd (Uganda Martyrs University – Faculty of Health Sciences) supervised the study and gave insight on data analysis and reporting. Dr Mugisha Noleb (Uganda Cancer Institute) provided guidance on the content of data collection tools and writing of this manuscript.

## Funding

This study was supported by Uganda Cancer Institute (UCI) and the African Development Bank (AfDB) through the UCI-AfDB Research and training grants.

## Research institution

Uganda Martyrs University and Uganda Cancer Institute.

## Competing interests

The authors have declared that no competing interests exist.

## Data availability statement

All relevant data are within the paper.

## Figures and Tables

**Table 1.** Demographic characteristics of study participants in Mayuge, Eastern Uganda.

Demographic characteristic		Frequency (*N* = 400)	Percentage (%)	Mean (SD)
Age in years	18–25	135	33.8	32.9(11.9)
	26–30	74	18.5	
	31–35	41	10.3	
	36–40	50	12.5	
	41–45	31	7.8	
	46+	69	17.3	
Marital Status	Single	95	23.8	
	Married/cohabiting	305	76.3	
Education Level	Primary	212	53.0	
	Post primary	188	47.0	
Ethnic Group	Bantu	356	89.0	
	Nilotics	33	8.3	
	Nile Hamites	9	2.3	
	Hamites	2	0.5	
Languages spoken	Monolithic(Lusoga only)	237	59.3	
	Linguistic	163	40.8	
Personal monthly income (Ug.Shs)	0–50,000	342	85.5	37,400(5,000)
	>50,000	58	14.5	
Employment	Formal	12	3.0	
	Informal	388	97.0	
Religion	Christians	192	48.0	
	Muslims	204	51.0	
	Others	4	1.0	

Source: primary data

**Table 2a.** Summary of the level of cervical cancer literacy among women in Mayuge-Eastern Uganda.

Cervical cancer literacy domains		Frequency (*N* = 400)	Percentage (%)	Mean(SD)
Cervical Cancer Awareness	Limited	362	90.5	36.35(21.22)
	Basic	21	5.3	
	Adequate	17	4.3	
Print literacy	Limited	310	77.5	51.85(24.13)
	Basic	70	17.5	
	Adequate	20	5.0	
Oral literacy	Limited	148	37.0	74.40(23.67)
	Basic	125	31.3	
	Adequate	127	31.8	
Numeral literacy	Limited	394	98.5	27.10(22.39)
	Basic	0	0.0	
	Adequate	6	1.5	
Perceived e-HL	Limited	361	90.3	23.72(26.96)
	Basic	13	3.3	
	Adequate	26	6.5	
Overall	Limited	387	96.8	42.68(12.23)
	Basic and adequate	13	3.3	

***Source:*** The limited level of functional cervical cancer literacy among women in Mayuge rural cancer registry population, Eastern Uganda: a multidimensional mixed method study.

**HL level cut-offs/classification: McCormack *et al* [9] cut-offs: Adequate = ≥82, Basic = 70–81 and limited = <70 for awareness, oral, print, perceived e-health and Weiss *et al* [25] cut-offs in the NVS: Adequate = 4–6 correct responses, Basic/low = 2–3 correct responses and Limited = 0–1 correct response. *** The agree and strongly agree and usefull/important and very usefull/important were scored as 1 and the rest as 0 to generate score for e-health.

**Table 2b.** Cervical cancer literacy assessment scorecard items.

HL domains assessed:	Score (%)	HL level cutoffs: McCormack *et al* [9] and Weiss *et al* [25] See footnote
**Domain1: cervical cancer awareness (**Prior knowledge): 1 = Correct response, 0 = Incorrect response		
What cancer is?	25.5	
What cervical cancer is?	40.5	
Risk factors of cervical cancer?	26.3	
Diseases and conditions that increase risk of developing cervical cancer?	27.8	
How HPV is transmitted?	33.8	
How to detect cervical cancer early?	37.5	
Purpose of a biopsy?	18	
Meaning of cervical cancer stages?	32.3	
Meaning of cervical cancer meta stasis?	33.8	
Whether early defected cervical cancer can be cured?	66	
Purpose of palliative care?	59.3	
**Domain1: cervical cancer Awareness average score (%)**	**36.4**	Limited literacy
**Domain2: cervical cancer oral literacy** (Listening with comprehension): 1 = Correct response, 0 = Incorrect response		
What was this audio message about?	78.8	
What have you learnt from this audio message?	71.6	
Recommended age for cervical cancer screening?	74.3	
How cervical cancer can be prevented?	73.5	
The benefit of early detection?	72.0	
**Cervical cancer oral literacy average score (%)**	**74.4**	Basic literacy
**Domain3: cervical cancer print literacy** (Reading with comprehension 1 = Correct response, 0 = Incorrect response		
What was this message about?	51.8	
What have you learnt from this message?	50.2	
What cervical cancer is?	51.0	
How is HPV transmitted?	55.5	
What are the ways of reducing the risk of developing cervical cancer?	51.8	
**Domain3: cervical cancer print literacy average score (%)**	**51.8**	Limited literacy
**Domain4: cervical cancer numeral literacy score** (Comprehension of numerical information in health messages): 1 = Correct response, 0 = Incorrect response		
Number of times a woman not living with HIV will screen for cervical cancer in a period of 10 years?	30.0	
Number of times a woman living with HIV will screen for cervical cancer in a period of 10 years?	30.0	
When to seek the next cervical cancer screening after testing negative today when living with HIV?	22.5	
What 5-year survival rate for cervical cancer means?	22.0	
Number of times a cervical cancer patient will receive radiation treatment when advised to receive the treatment five times per week from Monday to Friday in 4 weeks?	35.3	
Whether 90% of smokers will develop cervical cancer?	21.0	
**Cervical cancer numeral literacy average score (%)**	**27.1**	Limited literacy
**Domain5: cervical cancer perceived e-HL s**c**ore** (Perceived interest and ability to benefit from mobile phone health message platform). **In first two questions**: 1 = Not useful/important at all, 2 = Not useful/important, 3 = Unsure, 4 = Useful/important, 5 = Very useful/important. **In the rest of questions**: 1 = Strongly disagree, 2 = Disagree, 3 = Undecided, 4 = Agree, 5 = Strongly agree.		
How useful phone can help on making health decision?	59.3	
How important to access health information on a phone?	53.3	
I know what health resources can be accessed on phone?	12.0	
I know where to find health resources on a phone?	13.5	
I know how to find health information on a phone?	12.8	
I know how to use the phone to answer my question on health?	17.2	
I know how to use the health information I find on the phone?	14.3	
I have the skills I need to evaluate the health information I find on the phone?	15.7	
I can tell high quality health information from low quality health information?	19.5	
I feel confident in using health information from phone or internet to make health decisions?	15.8	
**Domain5: cervical cancer perceived e**-**HL score (%)**	**23.7**	Limited literacy
**Overall cervical cancer HL level** (Average of five domains)	**42.6**	Limited literacy

Source: primary data

**HL level cut-offs/classification: McCormack *et al* [9] cut-offs: Adequate = ≥82, Basic =70–81 and limited = <70 for awareness, oral, print, perceived e-health and Weiss *et al* [25] cut-offs in the NVS for numeracy: Adequate = 4–6 correct responses, Basic/low = 2–3 correct responses and Limited = 0–1 correct response. ***The statement agrees and strongly agrees and useful/important and very useful/important were scored as 1 and the rest as 0 to generate score for perceived e-health.

**Table 3.** Factors associated with cervical cancer literacy among women aged 18–65 years in Mayuge district.

Cervical cancer literacy					
Factors	Category	Limited *N* (%)	Not limited *N* (%)	OR (95% CI)	*p*-Value
Age in years	18–25	132(97.8)	3(2.2)	1.73(0.45–6.38)	0.556
	26 above	255(96.2)	10(3.8)		
Marital status	Single	90(94.7)	5(5.3)	0.49(0.16–1.52)	0.205
	Married	297(97.4)	8(2.6)		
Education level	Primary	209(98.6)	3(1.4)	3.91(1.06–14.44)	0.044**
	Post Primary	178(94.7)	10(5.3)		
Ethnicity	Bantu	343(96.3)	13(3.7)	0.96(0.84–0.98)	0.377
	Others	44(100.0)	0(0.0)		
Languages	Lusoga	231(97.5)	6(2.5)	1.73(0.57–5.24)	0.329
	Linguisitic	156(95.7)	7(4.3)		
Personal income	0–50,000	332(97.1)	10(2.9)	1.81(0.48–6.79)	0.414
	>50,000	55(94.8)	3(5.2)		
Occupation	Formal	10(83.3)	2(16.7)	0.15(0.03–0.746)	**0.054**
	Informal	377(97.2)	11(2.8)		
Religion	Christians	186(96.9)	6(3.1)	1.08(0.36–3.27)	0.892
	Others	201(96.6)	7(3.4)		
Radio ownership	Yes	276(95.5)	13(4.5)	0.96(0.93–0.98)	0.024**
	No	111(100.0)	0(0.0)		
Mobile phone ownership	Yes	246(95.7)	11(4.30	0.32(0.07–1.45)	0.149
	No	141(98.6)	2(1.4)		
Newspaper reading frequency	Never	314(97.5)	8(2.5)	2.69(0.86–8.46)	**0.079**
	Once a while	73(93.6)	5(6.4)		
Health facility visit during last 12 months	No	103(100.0)	0(0.0)	1.05(1.02–1.07)	0.025**
	Yes	284(95.6)	13(4.4)		
Absolute perceived risk of cervical cancer	Low	176(97.2)	5(2.8)	1.34(0.429–4.15)	0.617
	High	211(96.3)	8(3.7)		
Cervical cancer screening	Yes	95(99.0)	1(1.0)	3.90(0.50–30.42)	0.204
	No	292(96.1)	12(3.9)		
Routine cervical cancer screening provided	Yes	31(100.0)	0(0.0)	1.04(1.02–1.06)	0.611
	No	356(96.5)	13(3.5)		
HPV vaccination available	Yes	127(97.7)	3(2.3)	1.63(0.98–6.02)	0.560
	No	260(96.3)	10(3.7)		
Cervical cancer and other Cancer-related health education	Yes	54(94.7)	3(5.3)	0.54(0.14–2.03)	0.409
	No	333(97.1)	10(2.9)		

** Significant at 5% level

**Table 4.** The 3 × 3 table of PEN-3 categorised FGD Results among Mayuge women-Eastern Uganda.

**PEN-1 and PEN-2 and their applications**	**Positive beliefs and values:** Practices, values, beliefs, and relationships that produce positive cervical cancer outcome	**Existential beliefs and values:** Values and beliefs that do not have negative effect, but concerned with the nature of reality	**Negative beliefs and values:** Practices, values, beliefs, and relationships that produce negative cervical cancer outcome
**Perceptions:** The knowledge, attitudes, values and beliefs that influence cervical cancer prevention and control outcome	Not bothering to treat some disease early can make it change into cancer, Perceived danger of multiple sexual partners and giving births to many children, Perceived danger of poor personal hygiene		Cervical cancer has no cure Cervical cancer screening makes a woman infertile Cervical cancer treatment makes a women not to give birth Misconception that modern family planning causes cervical cancer
**Enablers:** Accessibility, availability, ease and cultural structures that may influence cervical cancer literacy-obtaining, decision and navigation to benefit from cervical cancer prevention and control interventions.	The value of obtaining cervical cancer treatment from hospital Viewing district health facility workers as valuable sources for cervical cancer information and screening service providers.		Lack of financial support from husband. Lack of and or low level of personal income makes women unable to access services Other competing priorities such as school fees denies them allocating resources health Low level of education makes it difficult to understand health information Mayuge lacks cervical screening and education cancer services Long waiting time to get screened at Mulago hospital and delay to obtain results when samples are removed. Long distance to Uganda cancer Institute-Mulago
**Nurturers:** Influence such as family, kin, peers, community members, beliefs, attitudes, media	Radio provides access to cervical cancer information, Viewing husband support as being important to nurture access to services.		Health facilities do not conduct cervical cancer education, Restriction from husband Domestic work takes most of their time, Radio talks and announcements are run fast making it difficult to understand the messages.

**Table 5.** Knowledge of cervical cancer preventive services available in Mayuge district health facilities and Regional hospitals-Eastern Uganda.

Cervical cancer preventive services available	Response	Frequency (*N* = 400)	Percentage (%)
Routine cervical cancer screening	Yes	31	7.8
	No	369	92.3
HPV vaccination for girls 10 years old or in P4 class	Yes	130	32.5
	No	270	67.5
Cervical cancer and other cancer-related health education	Yes	57	14.3
	No	343	85.8
Sexual and reproductive health services	Yes	120	30.0
	No	280	70.0
HIV counselling and testing	Yes	349	87.3
	No	51	12.8
Provision of free condoms at health facility	Yes	354	88.5
	No	46	11.5
Provision of free condoms through VHTs	Yes	144	36.0
	No	256	64.0
Provision of ART services in nearest health facility	Yes	317	79.3
	No	83	20.7

**Table 6.** Cervical cancer related risk behaviours among Mayuge women–Eastern Uganda.

Cervical cancer-related risky behaviours	Response	Frequency (*N* = 400)	Percentage (%)
Ever screened for Cervical Cancer	Yes	96	24.0
	No	304	76.0
Intention to screen (*n* = 340)	Yes	190	55.8
	No	150	44.1
Age at first sexual intercourse	< 18	343	85.8
	18 and above	57	14.3
Self-reported HIV status	Positive	86	21.5
	Negative	314	78.5
History of STIs other than HIV	Yes	202	50.5
	No	198	49.5
Number of lifetime sexual partners	One	15	3.8
	Multiple	385	96.3
History of sexual intercourse for money	Yes	33	8.3
	No	367	91.8
Frequency of condom use	Never	243	60.8
	Usually	157	39.3
Age when you had first pregnancy	< 20	329	82.3
	20 above	71	17.8
Parity	Less than 3	92	23.0
	3 above	308	77.0
History of tobacco use	Yes	28	7.0
	No	372	93.0
History of alcohol consumption	Yes	138	34.5
	No	262	65.5
Vaccinated against HPV	Yes	0	0
	No	400	100
History of modern contraceptive use (other than condom)	Yes	279	69.8
	No	121	30.3
